# Comparison of Different Tissue Clearing Methods for Three-Dimensional Reconstruction of Human Brain Cellular Anatomy Using Advanced Imaging Techniques

**DOI:** 10.3389/fnana.2021.752234

**Published:** 2021-11-11

**Authors:** Marina Scardigli, Luca Pesce, Niamh Brady, Giacomo Mazzamuto, Vladislav Gavryusev, Ludovico Silvestri, Patrick R. Hof, Christophe Destrieux, Irene Costantini, Francesco S. Pavone

**Affiliations:** ^1^European Laboratory for Non-linear Spectroscopy, University of Florence, Florence, Italy; ^2^Department of Physics and Astronomy, University of Florence, Florence, Italy; ^3^National Institute of Optics, National Research Council, Rome, Italy; ^4^Nash Family Department of Neuroscience, Friedman Brain Institute, Icahn School of Medicine at Mount Sinai, New York, NY, United States; ^5^UMR 1253, iBrain, INSERM, Université de Tours, Tours, France; ^6^Department of Biology, University of Florence, Florence, Italy

**Keywords:** clearing techniques, optical microscopy, immunofluorescence, light-sheet fluorescence microscopy, expansion microscopy

## Abstract

The combination of tissue clearing techniques with advanced optical microscopy facilitates the achievement of three-dimensional (3D) reconstruction of macroscopic specimens at high resolution. Whole mouse organs or even bodies have been analyzed, while the reconstruction of the human nervous system remains a challenge. Although several tissue protocols have been proposed, the high autofluorescence and variable post-mortem conditions of human specimens negatively affect the quality of the images in terms of achievable transparency and staining contrast. Moreover, homogeneous staining of high-density epitopes, such as neuronal nuclear antigen (NeuN), creates an additional challenge. Here, we evaluated different tissue transformation approaches to find the best solution to uniformly clear and label all neurons in the human cerebral cortex using anti-NeuN antibodies in combination with confocal and light-sheet fluorescence microscopy (LSFM). Finally, we performed mesoscopic high-resolution 3D reconstruction of the successfully clarified and stained samples with LSFM.

## Introduction

A deep understanding of the human brain’s structural and functional organization is of fundamental importance for biomedical studies. Different approaches can be used to study the anatomical features of the brain. Detailed, three-dimensional (3D) images of the human brain anatomy obtained using magnetic resonance imaging still lack cellular resolution (Despotović et al., 2015). This can be reached with classical histological techniques using thin slices ≤100 μm, highlighting issues related to the 3D reconstruction of extended parts of tissue up to the reconstruction of the whole brain (Amunts et al., 2013; Ding et al., 2015). In more recent years, major advances in clearing techniques (Costantini et al., 2019) and the advent of fast light-sheet fluorescence microscopy (LSFM) systems (Power and Huisken, 2017; Olarte et al., 2018; Hillman et al., 2019; Ueda et al., 2020a; Silvestri et al., 2021) have allowed the achievement of rapid 3D histology of whole organs up to imaging transparent rodent bodies (Pan et al., 2016; Kubota et al., 2017; Cai et al., 2019). However, human tissue transparency is extremely challenging due to the autofluorescence contributions in aged tissue (Ueda et al., 2020a,b; Pesce et al., 2021a). The adult human brain is characterized by the accumulation of intra- and extracellular molecules such as lipofuscin, neuromelanin pigments, and collagen, which produce a strong autofluorescence signal and prevent probe diffusion throughout the tissue (Monnier et al., 1984; Costantini et al., 2021a). Particularly, the presence of dense molecules creates a sturdy network, which hinders permeabilization of the tissue and reduces diffusion of standard antibodies (∼150 kDa) (Murray et al., 2015; Lai et al., 2018; Park et al., 2018; Ku et al., 2020; Zhao et al., 2020; Costantini et al., 2021b). Different strategies to increase the pore size of fixed specimens (Chung et al., 2013; Gleave et al., 2013; Lai et al., 2018; Wassie et al., 2019), as well as reducing non-specific antibody-samples interactions (Hama et al., 2015; Murray et al., 2015), and also increasing active transports by electrophoresis (Lee et al., 2016; Wang et al., 2018) can be used to reduce tissue density and improve probe penetration speed into large samples. Another important challenge in tissue staining is reaching a homogeneous antibody labeling against high-density epitopes (Chung et al., 2013; Ueda et al., 2020b; Pesce et al., 2021b). The best candidate in terms of distribution density in neuronal somata is the neuronal nuclear antigen (NeuN), which is widely used for quantitative neuronal morphometric studies of human brain tissue (Gittins and Harrison, 2004). Due to the limitations mentioned above, it is challenging to obtain a deep and uniform staining of thick human brain slices, leading to partial labeling of samples with consequent image interpretation errors.

Here, we tested different clearing protocols in combination with NeuN immunolabeling in human brain slices to perform volumetric reconstruction with advanced microscopy techniques. Indeed, the choice of the best clearing approach is strongly influenced by tissue characteristics, epitope preservation, and staining efficiency in the human samples. Also, such clearing methodologies require generating stable specimens to preserve the endogenous biomolecules and perform fluorescence retention following lipid removal. In this work, we employed four independent protocols optimized for human brain slices: CLARITY (Chung et al., 2013), SWITCH (Murray et al., 2015), SHIELD (Park et al., 2019), and expansion microscopy (ExM) (Chen et al., 2015). The CLARITY-based clearing procedure uses the paraformaldehyde-bridges to crosslink the endogenous proteins to a polyacrylamide meshgel, which allows successful imaging of human normal brain samples and in diseased conditions (Ando et al., 2014; Costantini et al., 2015; Liu et al., 2016; Phillips et al., 2016; Morawski et al., 2018). Instead, SWITCH and the last implementation SHIELD exploit the “system-wide control of interaction time and kinetics of chemicals (SWITCH)-off and -on buffer,” which allow a uniform and controlled crosslinking reaction of two different fixative molecules, glutaraldehyde (Murray et al., 2015; Costantini et al., 2021b) and a polyepoxy chemical (Park et al., 2019). This generates a chemical- and heat-resistant hybrid tissue/gel. On the other hand, ExM uses physical expansion to allow imaging of sub-diffraction biological features using conventional microscopes (Chen et al., 2015; Pesce et al., 2019; Wassie et al., 2019). The meshgel’s ability to retain the fluorescence signal and absorb water produces a 4-fold isotropic expansion in every dimension, which results in an excellent clearing and index-matching process of the hydrogel-embedded human samples.

## Materials and Methods

### Human Brain Specimens

Human tissue samples were procured by the body donation program (Association des dons du corps) of Université de Tours and from the Massachusetts General Hospital (MGH). Written consent was obtained from healthy participants prior to death, including the brain for any educational or research purposes. The authorization documents of the Association des dons du corps are kept with the Body Donation Program at the Université de Tours, with the MGH Autopsy Services in Boston, MA, United States, and are available upon request. Collected within the general frame of the approved IRB submission to the Partners Institutional Biosafety Committee (PIBC., protocol 2003P001937), the tissue samples given by MGH Autopsy Services do not require a specific Ethics Approval documentation.

Upon collection, samples were placed in neutral buffered formalin (pH 7:2–7:4) (Diapath, Martinengo, Italy) and stored at room temperature. Blocks from the fixed samples were washed with phosphate-buffered saline (PBS) solution at 4°C with gentle shaking for 1 month. Blocks were embedded in low melting agarose (4% in 0.01 M PBS) and cut into 500 ± 50 μm and 100 ± 10 μm coronal sections with a vibratome (Vibratome 1000 Plus, Intracel Ltd., United Kingdom). After cutting, the agarose surrounding each slice was removed. For this work, we used brain slabs of the precentral cortex from a 99-year-old subject (for the cell counting analysis and optimization of the clearing process), that was stored for 6 months in formalin, a Broca’s area brain slab from a 70-year-old subject stored in formalin for an unknown time, and a Broca’s brain slab from a 70-year-old subject stored for 2 years formalin.

### CLARITY Protocol for Human Brain Slices

CLARITY was performed in agreement with the protocol of Chung et al. (2013). Slices of 500 μm were mounted in a sandwich filled with a hydrogel CLARITY solution consisting of 4% (vol/vol) paraformaldehyde (PFA), 4% (vol/vol) acrylamide, 0.05% (vol/vol) bisacrylamide, 0.25% (wt/vol) VA044 in PBS. The sandwich is made by two coverslips separated by a laser-cut flat stainless steel spacer with the same thickness as the tissue slice (500 μm) (Supplementary Figure 1B). The sample was placed between the two coverslips, approximately in the middle of the spacer. The three pieces are held together by a two-component glue (Picodent twinsil speed). An opening along the shorter side of the spacer allowed the precise filling of the sandwich with the hydrogel solution using a syringe, taking care not to form bubbles, which would create breakpoints in the crosslinks formation. The sandwich was placed in a 50 ml container and soaked with the hydrogel solution. The specimens were incubated in the same solution at 4°C for 7 days. Afterward, the samples were degassed with nitrogen for 10 min and then moved to 37°C for the polymerization process. Next, the excess of the polymerized hydrogel was removed and the samples were cleared with a clearing solution [200 mM (wt/vol) boric acid, 4% (wt/vol) sodium dodecyl sulfate; pH 8.5] at 37°C under gentle shaking for 7–10 days. After the clearing process, brain slices were incubated in PBST (PBS and 0.1% Triton X-100, pH 7.4) at 37°C for 2 days to remove the SDS.

### SHIELD Protocol for Human Brain Slices

The SHIELD protocol was performed following the protocol Park et al. (2019). The slices were incubated for 24 h at 4°C with shaking for 1 day in the SHIELD-Off solution (10 ml SHIELD-Epoxy Solution, 5 ml SHIELD-Buffer Solution, 5 ml DI Water, LifeCanvas). The solution was then replaced with the SHIELD-On Buffer (LifeCanvas) and SHIELD-Epoxy Solution (LifeCanvas) in a 1:1 ratio (final volume of 40 ml) at room temperature (RT) with shaking for 1 day. Finally, the slices were cleared for 3–4 days at 55°C using 200 mM SDS, 20 mM sodium sulfite, 20 mM boric acid and washed three times in PBST at 37°C for 24 h.

### SWITCH Protocol for Human Brain Slices

The SWITCH protocol was performed following the protocol of Pesce et al. (2021b). The samples were incubated at 4°C for 24 h in 20 ml of SWITCH-Off solution, consisting of 50% PBS titrated to pH 3 using HCl, 25% 0.1 M HCl, 25% 0.1 M potassium hydrogen phthalate (KHP) and 4% glutaraldehyde. The human brain slices were then incubated at 4°C for 24 h in the SWITCH-On solution, containing PBS pH 7.4 with 1% glutaraldehyde. After three washes in PBS at RT 1 h each, the fixative reagent in the samples was inactivated by overnight incubation in a solution consisting of 4% glycine and 4% acetamide at 37°C. Next, the slices were washed four times (2 h each) and then incubated in the clearing solution containing 200 (wt/vol) mM SDS, 20 mM (wt/vol) sodium sulfite, 20 mM (wt/vol) boric acid for 2–4 days at 55°C depending on the sample size. After the clearing process, the samples were washed three times in PBST at 37°C for 24 h to remove SDS.

### Expansion Microscopy Protocol for Human Brain Slices

For the ExM experiments, the 100 μm-thick human brain slices were permeabilized in PBST for 2 h at 37°C. Next, the primary antibody was diluted in PBST (dilution 1:100) and incubated at 37°C for 3 days. After three washes of 30 min at 37°C, the secondary antibody conjugated to Alexa Fluor 488 was incubated for 24 h at 37°C. After three washes of 30 min at 37°C, and then three washes of 10 min at RT, the stained samples were functionalized overnight with the 10 mg/ml AcX (Tillberg et al., 2016) at RT in gentle shaking. After two washes of 15 min at RT, the functionalized samples were incubated in a gel solution consisting of 2 M (wt/vol) NaCl, 2.5% (vol/vol) acrylamide, 0.15% (vol/vol) N, N′-methylenebisacrylamide, 8.625% (wt/vol) sodium acrylate (SA), 0.01% (wt/wt) 4-hydroxy-TEMPO (4-HT), 0.2% (wt/vol) tetramethylenediamine (TEMED), and 0.2% (wt/vol) ammonium persulfate (APS) in distilled water, with the initiator APS added last, for ∼1 h at 37°C. The gelation process was carried out in a homemade chamber with a thickness of 200 μm, containing ∼50 μl of the gel solution (Pesce et al., 2019; Bianchini et al., 2021). The gelled specimens were soaked in the digestion solution consisting of 50 mM (vol/vol) Tris–HCl (pH 8), 1 mM (wt/vol) EDTA, 0.5% (vol/vol) Triton X-100, 1 M (wt/vol) NaCl, supplemented with 8 units/ml proteinase K added freshly for 24 h at 37°C with gentle shaking. Finally, the digested samples were transferred to a 60-mm Petri dish and soaked in distilled water for 2 h, with water changes every 30 min.

### Decolorization Treatments

To reduce autofluorescence in each clearing method, we tested two different treatments alone or in combinations (Pesce et al., 2021b). Here, we report the optimized protocols for each clearing method (Supplementary Figure 1A). Before starting the CLARITY protocol, we treated human brain slices with 30% (vol/vol) H_2_O_2_ diluted in DI water for 1 h at RT. After three washes in PBS (10 min each), samples were incubated with alkaline antigen retrieval solution (AR) consisting of 10 mM (vol/vol) Tris base, 1 mM (wt/vol) EDTA solution, 0.05% (vol/vol) Tween 20, pH 9 for 10 min at 95°C. This step increases the reaction sensitivity of antibodies directed to specific targets. The specimens were then cooled at RT for 30 min and washed in DI water 5 min each. Finally, the samples were equilibrated in PBS for at least 1 h at RT. For the SWITCH-processed slice, the decolorization protocol was performed according to the SHORT protocol (Pesce et al., 2021b). In particular, after performing SWITCH protocols, samples were treated with H_2_O_2_ and AR except that the clearing process was performed first. Finally, SHIELD-processed slices were treated with 15% (vol/vol) H_2_O_2_ diluted in DI water (1 h at RT) after the clearing process. Immunostaining was performed following all treatments.

### Immunostaining and Clearing of Human Brain Slices

After clearing, the samples were incubated with the primary antibodies for 5 days in PBST. After three washes in PBST (1 h each), stained slices were incubated for 3 days with the secondary antibody in PBST. After, the samples were extensively washed with PBST for 24 h. Each step was performed at 37°C for SWITCH and CLARITY, while at 4°C for SHIELD according to the published protocols (Pesce et al., 2021b). Specific primary and secondary antibodies for this study were presented in Table 1. Stained samples were then cleared in increasing concentration of 2,2′-thiodiethanol (TDE) in 0.01 M PBS. The first incubation at 30% (vol/vol) TDE/PBS was performed for 2 h at RT, while the final equilibration in 68% (vol/vol) TDE/PBS was reached by incubating the sample overnight at RT with gentle shaking. The slices were then placed in a sandwich holder with a quartz coverglass to match the refractive index (RI) of 68% TDE (1.46), filled with 68% TDE/PBS and acquired with the different microscopic techniques.

**TABLE 1:** Primary and secondary antibodies tested in cleared human brain slices and used dilutions.

**Molecule**	**Company**	**Cat. N.**	**Host**	**P/M**	**Dilution**
NeuN	St. John’s Laboratory	STJ113146	Rabbit	M	1:200
NeuN	ProteinTech	26975-1-AP	Rabbit	P	1:200
NeuN	Arigobio	ARG52283	Mouse	M	1:200
NeuN	Arigobio	ARG10712	Rabbit	P	1:200
NeuN	Abcam	ab104224	Mouse	M	1:200
NeuN	Merck	ABN78	Rabbit	P	1:200
NeuN	Abcam	ab207282	Rabbit	M	1:200
NeuN	Abcam	ab134014	Chicken	P	1:200
NeuN	Merck	ABN91	Chicken	P	1:100
NeuN	Cell Signaling	D4G40	Rabbit	M	1:200
Non-phosphorylated neurofilament proteins	Eurogentec	SMI-32P	Mouse	M	1:200
β-tubulin	ProteinTech	66375-1-Ig	Mouse	M	1:200
Neurofilaments	Abcam	ab8135	Rabbit	P	1:200
Anti-Rabbit IgG, AF 568	Abcam	ab175470	Donkey	P	1:200
Anti-Mouse IgG, AF 568	Abcam	ab175700	Donkey	P	1:200
Anti-Chicken IgY, AF 568	Abcam	ab175711	Goat	P	1:200
Anti-Chicken IgY, AF 647	Abcam	ab150171	Goat	P	1:500

### Confocal Imaging of Brain Slice

To examine the immunostaining quality, the slices were placed on a coverslip and imaged with a commercial confocal microscope: Nikon Eclipse TE300, with the Nikon C2 scanning head, using the Nikon Plan EPO 60× objective (numerical aperture 1.4, oil-immersion). The 488 and 568 nm lasers were used to excite Alexa Fluor 488 (ExM) and Alexa Fluor 568 (CLARITY, SHIELD, and SWITCH), respectively.

### Light-Sheet Fluorescence Microscopy

A custom-made inverted light-sheet microscope was used to image the brain slices (Pesce et al., 2020). Two identical and orthogonal objectives (LaVision Biotec LVMI-Fluor 12x PLAN with numerical aperture 0.53, WD 10 mm, magnification 12×) were used to alternatively excite and collect the fluorescence signal. The objectives have a correction collar for refractive index matching with the immersion solution (*n* = 1.46 for the tissue transformation protocols, *n* = 1.33 for expanded tissues). The scanned illumination setup and the sample stage assembly were custom-designed. For the fluorescence excitation pathway, two different excitation laser lines at either 488, 561, and 638 nm were employed simultaneously (Cobolt MLD 488, DPL 561 nm, and MLD 638 nm). An acousto-optical tunable filter (AAOptoelectronic AOTFnC-400.650-TN) modulates the transmitted power and wavelength, while a galvo mirror (Cambridge Technology 6220H) sweeps it across the detection focal plane, generating a digitally scanned light sheet. The objective lens collected the induced fluorescence onto a Hamamatsu ORCA Flash4.0v3 sCMOS camera, working in confocal detection mode (Baumgart and Kubitscheck, 2012; Gavryusev et al., 2019; Ricci et al., 2020). The emitted fluorescence signal was collected with a matched bandpass filter (Semrock FF03-525/50, FF01-600/52, or FF01-697/70). The sample was imaged by translating it along the horizontal direction in a snake-like pattern. To match the refractive index of the transformed tissues equilibrated in the TDE/PBS solution, 250-μm-thick fused-silica (*n* = 1.46) were used in a sandwich holder as described by Pesce et al. (2021b). For the expanded tissues, we used polytetrafluoroethylene (*n* = 1.34) which shows a compatible refractive index with water. To visualize the reconstruction of an entire acquired slice, a data translation is applied to the acquired images to compensate for the motion of the stage. The image is then rotated by 45° allowing the visualization of the acquired volume in the sample’s coordinate system. To stitch together all the acquired stacks, a custom- made stitching software called ZetaStitcher (GM, ‘‘ZetaStitcher: a software tool for high-resolution volumetric stitching’’)^1^ was used. The resulting data is spatially down-sampled at 3.3 μm^3^ isotropic resolution.

### Data Analysis

Plot intensity profiles were produced using Fiji (Schindelin et al., 2012). We selected a region of interest (ROI 800 × 1,300 μm^2^) from the whole images and we processed a Z projection of the entire thickness of the sample. We calculated the average intensity and the standard deviation (SD) and plotted the normalized values using OriginPro 9.0 (OriginLab Corporation). The neuronal counting for each clearing treatments (SWITCH and SHIELD) was performed by Fiji, using the plugin Analyze Particles. Three ROIs of 154,880 μm^2^ were selected at different penetration depths (0–160, 170–330, and 340–500 μm) for 6 random stacks (total 18 ROIs covering an area of 2.79 mm^2^). A binary mask (black and white) for the gray matter of each sample was manually set. To remove potential artifacts during the neuronal identification, areas lower than 40 μm^2^ and higher than 600 μm^2^ were discarded. The significance was determined using the two-samples *t*-test. A *P*-value of <0.05 is considered as indicative of a statistically significant difference between means (^∗∗^*P* < 0.01). Data are expressed and plotted as mean ± SD obtained from the same depth of several stacks. Signal to background (S/B) analysis was performed selecting a ROIs of 10 × 10 μm^2^ in the NeuN-labeled cellular soma and in the adjacent background by using Fiji. Four different high-resolution stacks of LSFM reconstructions in the gray matter were randomly selected for both SWITCH and SHIELD treated slabs. S/B of five neurons was calculated along three depths of the stack (0–50, 225–275, and 450–500 μm; *n* = 20 neurons for each depth). We plotted mean ± SD using OriginPro 9.0. For the expanded tissue, the expansion factor was calculated using Fiji by measuring the sample thickness of the pre- and post-expansion samples (*n* = 10). For the signal-to-noise ratio analysis of NeuN staining, 16 ROIs of 2 × 2 μm^2^ for SWITCH-processed slices and pre-expansion tissues, as well as the 8 × 8 μm^2^ (*n* = 8 for signal and 8 for background) for post-expansion tissue were selected by Fiji and plotted (mean ± SD) using OriginPro 9.0.

## Results

### Comparison of Different Clearing Methods

**FIGURE 1:**
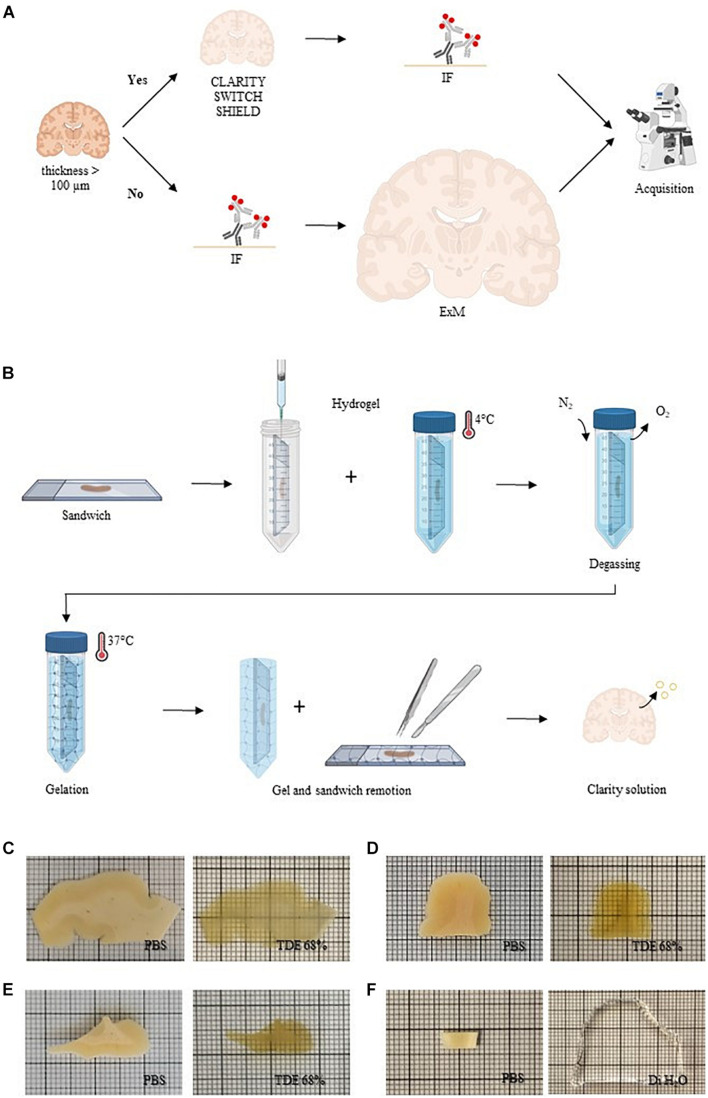
Sample processing. **(A)** Conceptual diagram of the sample processing pipeline. Human brain slices with a thickness greater than 100 μm were cleared with different clearing protocols (CLARITY, SWITCH, and SHIELD) and then immunolabeled (IF, immunofluorescence). Slices with a thickness less than 100 μm were stained before to perform ExM. All the samples were acquired with different optical techniques. Created with BioRender.com. **(B)** CLARITY sandwich preparation for hydrogel inclusion. Human brain slices were placed on two coverslips, separated by a 500 μm-thick stainless steel spacer. The sandwich was then filled with a hydrogel CLARITY solution using a syringe and placed in a 50-ml tube dipped with the same solution and incubated at 4°C. After the samples were degassed with nitrogen (N_2_) removing the oxygen (O_2_). The temperature was increased to 37°C to initiate polymerization. Finally, the embedded sample was extracted from the gel and washed with a clearing solution to remove lipids. Created with BioRender.com. **(C–F)** Representative images of human brain slices pre-clearing (in PBS) and after refractive index matching in 68% TDE: CLARITY **(A)**, SWITCH **(B)**, and SHIELD **(C)** and dH_2_O (ExM) **(F)**.

We tested different clearing methods on human brain slices with the aim to find the best protocol compatible with NeuN staining. To enhance tissue transparency, we treated post-mortem formalin-fixed human brain slices of 500 μm of thickness with CLARITY (Chung et al., 2013), SWITCH (Murray et al., 2015), and SHIELD (Park et al., 2019; Figure 1A and Supplementary Figure 1A). The common concept behind these clearing methods is to stabilize the endogenous proteins and nucleic acids using appropriate fixative molecules before delipidation. After sample fixation, such methodologies require hydrogel formation, lipid extraction, and, after staining, refractive index matching of the samples. SWITCH and SHIELD use, respectively, glutaraldehyde and polyglycerol-3-polyglycidyl to generate a heat- and chemical-resistant hybrid tissue/gel. The slow diffusion and the surface accumulation of such fixatives require a step of suppression of the crosslinking reaction using the SWITCH-off buffer. Then, the specimens were transferred to the SWITCH-on solution to turn-on the fixative reaction. In contrast, the CLARITY protocol uses an acrylamide-based hydrogel to bind molecules with amino ends. A side effect of this step is the extensive presence of a cross-linking network surrounding the specimens, generated by the polymerization of polyacrylamide gels. After the polymerization process, it is necessary to remove the excess of gel to proceed with the subsequent treatments (e.g., staining and samples assembly), preserving the tissue architecture. However, removing gel in a relatively thin tissue slice is a very delicate process that can lead to the breaking of the sample. For this reason, we developed a sandwich method to eliminate issues related to gel overabundance especially on the slice’s surface (Figure 1B and Supplementary Figure 1B). Using this configuration, the brain slices were mounted between two glasses, which stabilizes the slices during the incubation in the hydrogel solution, and helps to remove the excess of the polymerized hydrogel surrounding the specimens. The subsequent lipids washout provided a high level of transparency in the sample including heavily myelinated white matter (Figure 1C). We also tested the SWITCH and SHIELD protocols that both yielded uniform clearing of the white and gray matter (Figures 1D,E). These clearing protocols optimized for the human brain showed an efficient clearing process in the white and gray matter of the tissue, demonstrating the compatibility of SHIELD protocol with the TDE refractive index matching medium (Costantini et al., 2015; Pesce et al., 2021b). In parallel, we assessed ExM as a clearing protocol variant (Chen et al., 2015; Gao et al., 2017), to investigate 100 μm-thick slices (Figure 1A and Supplementary Figure 1A). Similar to CLARITY, the ExM hydrogel is composed of an acrylamide/bisacrylamide backbone containing sodium acrylate, which allows water absorption through osmotic force, causing swelling. In contrast to the tissue transformation described above, ExM requires the labeling and the covalent functionalization of the specimens with opportune handles before the clearing process (Wassie et al., 2019). Such handles act as bridges between the fluorescent markers and the hydrogel synthesized throughout the specimens. After gel polymerization, the embedded samples were enzymatically digested. Finally, the specimens were soaked in distilled water (DiH_2_O) for achieving an isotropic 4-fold expansion (Figure 1F). In contrast to SWITCH, SHIELD, and CLARITY, which use the TDE/PBS solution for matching the RI of the delipidated samples (1.46), the expanded sample is 99% water, with a RI of 1.33.

### Assessment of NeuN Staining

**FIGURE 2:**
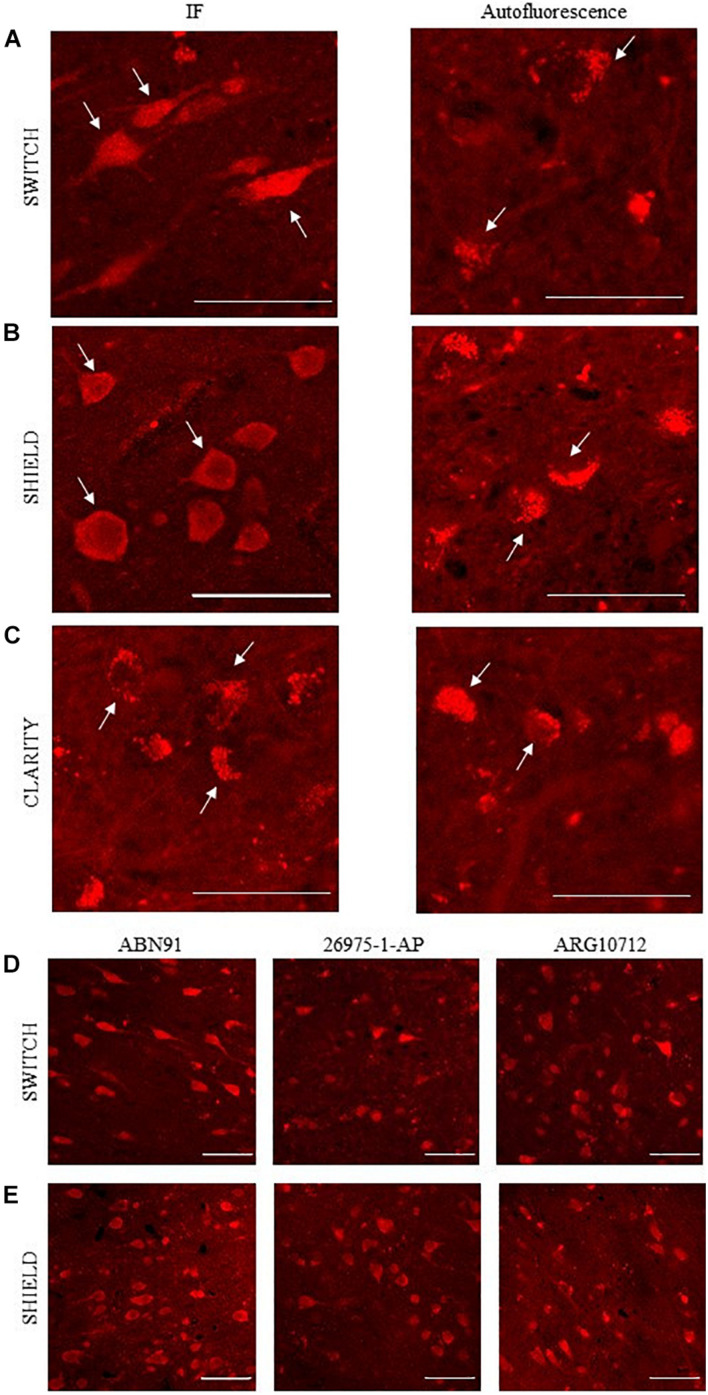
NeuN antibodies validation using confocal microscopy. **(A–C)** Representative confocal images of human brain slices stained using anti-NeuN antibody (ABN91; left): SWITCH **(A)**, SHIELD **(B)**, and CLARITY **(C)** samples, respectively. White arrows highlight three different stained neuronal bodies. On the right, autofluorescence signal of lipofuscin (white arrows). Excitation light, 568 nm; laser power, 1 mW. Objective lens, 60×; NA, 1.4. Scale bar = 50 μm. **(D,E)** Representative confocal images of SWITCH **(D)** and SHIELD **(E)** clarified human brain slices stained using ABN91, ARG 10712, and 26975-1-AP anti-NeuN antibodies. Scale bar = 50 μm.

Immunohistochemical analyses demonstrated the exclusive expression of NeuN protein in neurons (Gusel’nikova and Korzhevskiy, 2015) pointing out NeuN as an elective marker to get a general overview of neuron density in the brain. To evaluate the compatibility of NeuN immunostaining with different clearing methods, several anti-NeuN antibodies were tested (see Table 1). After staining, NeuN-immunoreactive neurons showed whole-body homogeneous staining, while the signal from lipofuscin appeared granular and confined in a small part of the neuronal body (Pesce et al., 2021a; Figures 2A–C). Direct and indirect immunofluorescence combined with confocal microscopy was used to examine NeuN immunostaining in SWITCH-, SHIELD-, and CLARITY-processed specimens. As the excitation line 405 and 488 nm show spurious and diffuse fluorescence signals due to the fixative (i.e., glutaraldehyde) and cofactor molecules (Monici, 2005; Lee et al., 2013), we optimized the staining protocol using Alexa 561 and the addition of H_2_O_2_ for decolorization (Pesce et al., 2021b). Results of labeling for each clearing method using anti-NeuN antibodies are reported in Supplementary Table 1. We found that only ABN91, 26975-1-AP, and ARG10712 are compatible with the SWITCH and SHIELD methods (Figure 2). Unfortunately, NeuN immunostaining was not suitable with the CLARITY protocol, even using antigen retrieval treatment, as we detected only the red-shifted lipofuscin signal (Figure 2C). To verify the robustness and the epitope preservation of CLARITY-processed specimens, we probed other neuronal markers (β-tubulin and neurofilament proteins), obtaining homogeneous staining of these cellular markers (Supplementary Figure 2), suggesting a possible loss or alteration of the NeuN antigen during the CLARITY protocol.

### Light-Sheet Fluorescence Microscopy Volumetric Imaging of NeuN-Immunolabeled Human Brain Slices

**FIGURE 3:**
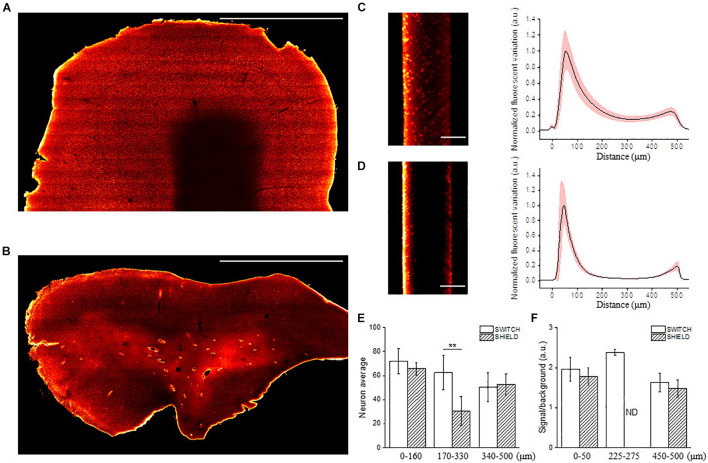
Representative LSFM images of clarified human brain slices labeled with different NeuN antibodies. Mesoscale reconstruction of SWITCH- **(A)** and SHIELD- **(B)** processed slices stained with anti-NeuN antibody ABN91 with Alexa Fluor 568. Excitation light 561 nm, laser power 5 mW. Maximum intensity projection (MIP) within 60 μm at 150 μm depth. Scale bar = 1 mm. **(C,D)** Lateral reslicing of SWITCH **(C)** and SHIELD **(D)** processed samples with corresponding normalized plot intensity profiles (black line: mean; light-red outline: standard deviation). Scale bar = 250 μm. **(E)** The graph shows the neuron quantification in the prefrontal cortex of two subsequent slabs of the same subject using SWITCH and SHIELD clearing techniques. The counting was performed using 3 ROIs selected at different penetration depths (0–160, 170–330, and 340–500 μm) for 6 random stacks (total 18 ROIs) in the gray matter. Data are reported as mean ± SD and two-sample *t*-test performed (**P < 0.01). **(F)** The column plot shows the signal to background ratio of neurons at different depths (0–50, 225–275, and 450–500 μm). It was not possible to identify labeled neurons in the center of the SHIELD-slabs, ND, not detectable.

**FIGURE 4:**
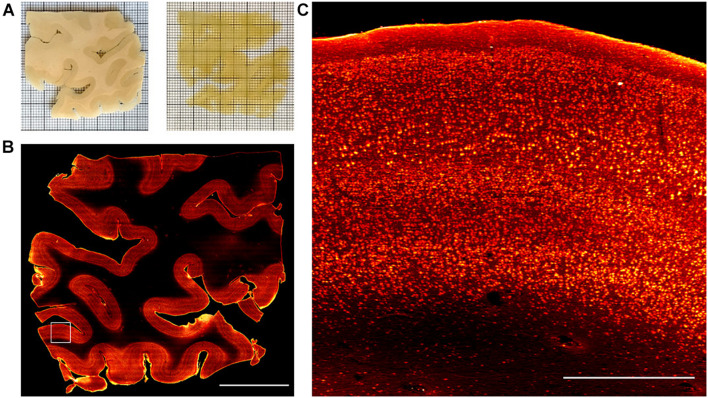
Representative LSM Broca’s area images. **(A)** Broca’s area images of human brain slices in PBS (pre-clearing) and after refractive index matching in the 68% TDE/PBS solution. **(B)** SWITCH-processed slice stained with anti-NeuN antibody. MIP of 30 μm at 250 μm depth. Scale bar = 1 cm. **(C)** High-resolution insert corresponding to the white square (3 mm × 3 mm) in **(B)**. Scale bar = 1 mm.

Once the three anti-NeuN antibodies suitable with SWITCH and SHIELD protocols were identified, we decided to use the chicken anti-NeuN antibody (ABN91) to perform volumetric staining of human brain slices. Antibodies produced in chicken are less common than those produced in rabbit (Weber et al., 2017), opening the possibility of performing multiple staining on the same slices to detect different markers. To verify the labeling efficiency throughout the thickness of the tissue slab, we imaged the labeled samples with LSFM. Figures 3A,B show the 3D-LSFM-reconstructions of SWITCH- and SHIELD-cleared tissues, respectively. To determine the quality of the staining, we observed the global fluorescence Z profile and we detected a decrease in the signal intensity from the surface to the middle of the sample thickness for both of these clearing methods (Figures 3C,D). In particular, the normalized intensity profiles of both methods show a peak in the first 100 μm, that drops down to 15% of the signal in SWITCH and 3% in SHIELD at 300 μm of depth. An increase of signal is observed from 450 to 500 μm of 23 and 10%, respectively, in SWITCH and SHIELD. To characterize this effect, we performed a signal recognition using the Analyze particles Fiji’s plugin. This automatic analysis employs a threshold to discriminate signals inside the image, yielding counts of those signals as an output. In our images, the fluorescent signal is emitted by cell bodies, and as such results in an indirect cell count. We analyzed two consecutive slabs in the prefrontal cortex of the same human brain sample immunostained for NeuN, treated with either SWITCH and SHIELD. We counted cells in the gray matter from 6 stacks selecting from each stack 3 ROIs at different depths (0–160, 170–330, and 340–500 μm) obtaining a significant difference in cells counting in the center of the slabs (Figure 3E). In particular, at this depth SHIELD showed a lower count (30.5 ± 12.1) compared to SWITCH (62.5 ± 14.2; *P* < 0.01) while, near to the surface of the samples, there is not difference in cell counting for both treatments. However, this automatic analysis does not permit the discrimination of the NeuN specific signals from the lipofuscin pigments signals (very dense in this specimen). To obtain a more comprehensive evaluation of the presence of antibody labeling deep inside the tissue, a manual analysis of the signal-to-background (S/B) of neurons at different depths was performed. Four different high-resolution stacks of LSFM reconstructions were randomly selected in the gray matter of the two samples and the S/B ratio of five neurons along three depths of the stack (0–50, 225–275, and 450–500 μm) were calculated (Figure 3F). The S/B was not detectable (ND) in the center of the SHIELD’s stacks, suggesting absence of specific labeling in this region, while the S/B ratio was comparable for both methods near the surface of the stack. Based on these results, SWITCH resulted to be the best choice for 500 μm-thick slabs. Thus, we tested our staining protocol on a 2 cm × 2 cm brain slice from Broca’s area of 500 μm thickness that was NeuN-immunolabeled using a goat anti-chicken Alexa Fluor 568-conjugated secondary antibody. Supplementary Figure 3A shows the result of the clearing protocol after the refractive index matching in 68% TDE. The reached transparency allowed us to perform volumetric reconstruction of the entire slice with LSFM with an isotropic resolution of 3.3 μm (Supplementary Figure 3B). We further employed this protocol on a larger slice (4 cm × 4 cm) to demonstrate the versatility of the method (Figure 4A). Figure 4B shows the slice reconstruction with an insert highlighting the pattern of the cortical layering (Figure 4C). For this application we used a secondary antibody conjugated to Alexa Fluor 647 to improve the contrast of the staining, as the background signals of the tissue are reduced at this wavelength (Pesce et al., 2021b). The calculated global fluorescence Z profile at 300 μm resulted in a 34% of the signal, showing a 10% increase compared to the signal obtained using Alexa Fluor 568 (Supplementary Figure 4).

### Mesoscopic Reconstruction of Expanded Human Brain Tissue

**FIGURE 5:**
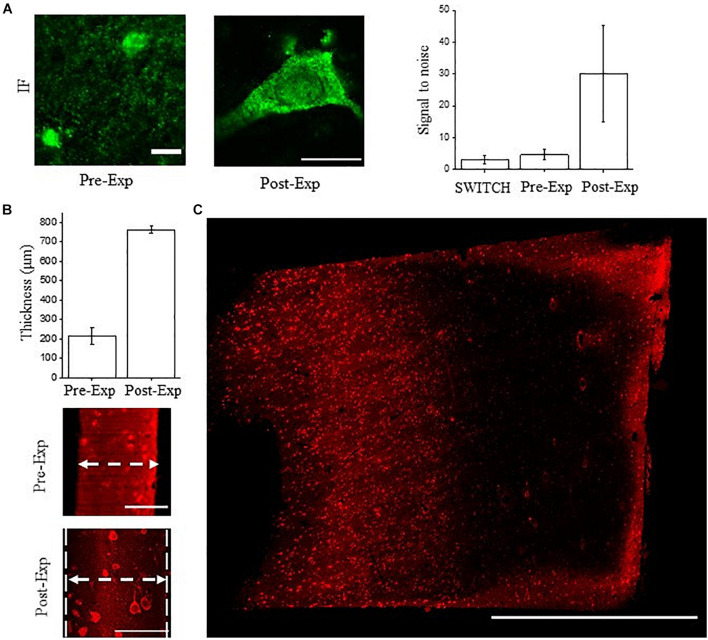
NeuN immunostaining validation using ExM. **(A)** Confocal images of pre- and post-expanded human cortex immunostained for NeuN (antibody ABN91) with Alexa Fluor 488. Objective lens, 60×; NA, 1.4; excitation light, 488 nm. Signal-to-noise ratio comparison between the tissue transformation protocol SWITCH, pre-expansion (classic immunofluorescence protocol), and post-expansion specimens. A significant improvement is observable in the post-expanded samples, imputable to the dilution of the non-specific signal and reduction of the autofluorescence. The choice of the fluorescent dyes and the acquisition setting were optimized for each treatment and clearing procedures. Pre-expansion scale bar = 10 μm; post-expansion scale bar (40/4) = 10 μm. **(B)** Expansion factor characterization using the sample thickness before (∼100 μm) and after expansion (∼400 μm) acquired by LSFM (*N* = 10). Pre-expansion scale bar = 100 μm; post-expansion scale bar (400/4) = 100 μm. **(C)** Maximum intensity projection of post-expanded human superior frontal cortex using our custom-made LSFM. Excitation light, 488 nm; power, 5 mW. Scale bar = 1 mm. The white arrows show the sample thickness used for quantifying the expansion factor.

We tested the compatibility of NeuN immunostaining with ExM in human brain slices. ExM uses classic immunofluorescence staining in relatively thin sections, and is challenging in human samples. ExM-processed tissues are less autofluorescent compared to those treated with SWITCH due to the absence of additional crosslinking agents like glutaraldehyde, and the excellent fluorescence retention by treating the sample with the AcX handle (Tillberg et al., 2016; Shen et al., 2020). For these reasons, we labeled NeuN with Alexa Fluor 488 in the ExM experiments. As shown in Figure 5A the anti-NeuN antibody (ABN91) shows efficient detection of neurons using confocal microscopy, with a S/B ratio similar to SWITCH. After gelation and digestion, the post-expanded sample was acquired by confocal microscopy, showing a high fluorescence retention of Alexa Fluor 488 and signal-to-noise ratio.

However, volumetric reconstruction of expanded human brain tissue using laser-scanning confocal microscopy is challenging due to the slow acquisition rate and photobleaching effect. Considering a 4-fold expansion factor with an initial sample volume of 3 mm × 3 mm × 0.1 mm, following ExM treatments, it would be transformed to a volume of 12 mm × 12 mm × 0.4 mm. For these reasons, we coupled ExM with LSFM (thereafter ExLSFM) (Bürgers et al., 2019; Düring et al., 2019) to quantify the expansion factor (Figure 5B), validate the probe penetration before the expansion process (Figure 5B), and acquire a large portion of expanded tissues (Figure 5C). First, using a permeabilization step in PBST and incubating the NeuN antibody at 37°C, we confirmed a full NeuN immunoreactivity distribution throughout the 100 μm-thick slices. After tissue expansion, we efficiently detected the neuron throughout the slices, by exploiting the resolution and signal-to-noise ratio improvement. Next, using the tissue thickness as a ruler, we quantified the expansion factor of human brain slices. After 24 h in the digestion buffer, the proteinase action and protein denaturation allow an isotropic meshgel expansion in the white and gray matter of 3.5 ± 0.8 times (expansion factor quantification, *N* = 10; Figure 5B). Such results demonstrate the compatibility of ExLSFM for investigating the human brain cytoarchitecture. Finally, we performed volumetric acquisition of the expanded human cortex (Figure 5C), achieving a lateral optical resolution of ∼350 nm (optical resolution of our custom made LSFM: 1.1 μm divided by the expansion factor 3.5).

## Discussion

Adult human brain tissue is challenging to clear due to the age-dependent accumulations of intracellular and extracellular pigments and long formalin fixation time (Monnier et al., 1984; Moreno-García et al., 2018, 2021). In recent years, various clearing methods have been developed to investigate the 3D molecular organization and the cellular architecture of different organs, including the brain. However, the optimization of these methods in terms of decolorization, transparency, and labeling efficiency, is still in progress. The long fixation and tissue pH variation can determine protein rearrangement, which may induce epitope masking effects and/or antigen damage (Scalia et al., 2017). The clearing methods employed in this study were developed mainly for animal models application, in particular mouse brain clearing. The possibility of coupling whole-brain clearing with fast imaging with LSFM has opened the possibility to study not only the cytoarchitecture of the brain (Silvestri et al., 2021), but also investigating neuronal activation using transgenic animals expressing immediate early genes (Franceschini et al., 2020). Many applications (Ueda et al., 2020b; Weiss et al., 2021) have promoted the field of tissue clearing, prompting scientists to obtain higher transparency and labeling. Nevertheless, animal models have important advantages compared to human tissues: there is no variability of post-mortem fixation conditions, blood is washed out from vessels, there are no autofluorescence signals from lipofuscin-type pigments, and, finally, they can express endogenous labeling. The human brain, however, requires specific clearing protocol optimizations, and more importantly, specific exogenous labeling conditions, to overcome the inherent limitations of the tissue. Indeed, as demonstrated here, antigen alteration and/or tight meshgel net prevent reliable immunostaining detection deep inside the tissue. Moreover, obtaining a perfect staining of generic markers such as NeuN in the whole thickness of the sample is a crucial step to achieve a precise structural map of neurons in the brain. However, highly expressed epitopes are difficult to label due to the diffusion of antibodies from the surface to the center of the slice: the binding of the antibodies to the first epitopes near the surface of a slice creates an obstacle which further reduces the penetration of more antibodies toward the center.

In this study, we compared different tissue transformation clearing techniques to understand the best protocol to perform labeling against high-density epitopes (Ueda et al., 2020b), in human brain slices. CLARITY (Chung et al., 2013), SWITCH (Murray et al., 2015), SHIELD (Park et al., 2019), and ExM (Chen et al., 2015) were used to generate transparent samples. CLARITY and ExM use an acrylamide/bisacrylamide backbone to generate a hybrid tissue/gel. The key differences for achieving a swellable hydrogel in ExM is the sodium acrylate incorporation into the gel and the digestion process. Also, ExM requires a small volume of hydrogel and 4-HT for the monomer diffusion throughout the specimens before the polymerization reaction (Tillberg et al., 2016), reducing the hydrogel amount. Instead, CLARITY requires a generous volume of the hydrogel solution and long incubation times for PFA bridges formation, causing an overabundance of polymerized gel around the sample. Removing the gel excess from the processed tissue is challenging, especially due to the fragility of the tissue during the extraction step. To overcome this limitation, we developed a “sandwich” method, which helps preserving the tissue architecture and produces a flattened sample without gel surplus. In addition, our data demonstrates that all clearing approaches showed a high transparency level, suggesting that the clearing protocol is not a discriminatory parameter, but it requires a molecular validation by immunofluorescence after the clearing process.

To address this issue, our choice was focused on NeuN immunostaining combined with advanced optical techniques, using confocal and LSFM. The NeuN protein is localized in most neurons in the central nervous system of mammals (Korzhevskii et al., 2009; Verdiev et al., 2009) and, for this reason, it is used as a pan-neuronal marker (Mullen et al., 1992). Although we tested several anti-NeuN antibodies in humans, only a few of them were compatible with our clearing procedures. In particular, three antibodies (26975-1-AP, ARG10712, and ABN91) allowed an efficient detection of neurons in SWITCH- and SHIELD-processed slices by confocal microscopy evaluation. Unfortunately, we did not find any anti-NeuN antibody compatible with CLARITY, probably due to an epitope-masking effect of the polyacrylamide gel as well as antigen damage during the processing. However, we demonstrated the robustness of the hybrid tissue/gel by successfully staining two other neuronal markers, β-tubulin and neurofilaments, in CLARITY-processed human brain slices.

Because confocal microscopy is limited by penetration depth, photobleaching and volumetric acquisition speed, we then imaged NeuN-immunostained samples using LSFM (Huisken and Stainier, 2009), demonstrating an efficient probe penetration throughout the thickness in SWITCH-processed slices compared to SHIELD. Our optimized protocol was used to reconstruct volumetric human brain slices with LSFM, demonstrating the reliability of the immunoreaction and reliability for application to large-scale analyses.

Finally, we characterized the NeuN immunostaining efficiency in ExM. ExM allows the enhancement of the signal-to-noise ratio and the final optical resolution by absorbing water, expanding the specimens 4-fold (Wassie et al., 2019; Parra-Damas and Saura, 2020). To obtain homogenous labeling, we performed immunofluorescence in 100 μm-thick human brain slices before expansion, which constrained the total observed sample volume. However, the native proteins were digested by proteases prior to expansion and, hence, could not be probed directly after expansion or performing multiple rounds of staining and stripping (Murray et al., 2015). In the future, our ExM protocol will be adapted to other variants, such as Magnified Analysis of the Proteome (MAP) (Ku et al., 2016) or miriEx (Shen et al., 2020), which preserves the endogenous proteins, allowing to perform the staining protocol after the expansion process.

All methods addressed in this work involve comparable physical principles despite different chemical reagents applied for the tissue transformation process. CLARITY has been successfully applied on mouse brains, but proved to be challenging on human brains. In fact, our results show a masking effect or epitope loss for the NeuN protein in CLARITY-processed tissue, inhibiting a successful mesoscopic reconstruction of such samples. Moreover, it requires specific equipment for the degassing needed to obtain gel polymerization, while SWITCH and SHIELD leverage glutaraldehyde and epoxy-resin to stabilize the tissues using simple laboratory equipment, making tissue preparation less challenging. Nevertheless, a drawback of SHIELD is the use of specific commercial resins that need to be purchased and last only a few months, requiring careful planning of experiments. In conclusion, to use CLARITY, we suggest testing different antibodies to evaluate the markers detection performances. For studies performed on thin slices (<150 μm) it is possible to use both SWITCH and SHIELD. For thicker slabs, we suggest using SWITCH as it has demonstrated reliable staining up to 500 μm, while in SHIELD it drops down very quickly. A possible explanation is that the net created by the epoxy resin is too tight, preventing good penetration of the antibodies deep inside the tissue. We believe that our work will help to perform structural imaging of such complex specimens efficiently using high-throughput imaging techniques such as LSFM.

## Data Availability Statement

The original contributions presented in the study are included in the article/Supplementary Material, further inquiries can be directed to the corresponding author/s.

## Ethics Statement

The studies involving human participants were reviewed and approved by the Body Donation Program (Association des dons du corps) of Université de Tours and by the body donation program from the Massachusetts General Hospital (MGH). The patients/participants provided their written informed consent to participate in this study.

## Author Contributions

MS, IC, and LP conceived of the study, performed the tissue transformation protocol, and wrote the first draft of the manuscript. LP and MS performed the ExM experiments. LP, MS, NB, and VG performed the imaging. MS, LP, and GM performed the data analysis. CD fixed and dissected the human brain samples. IC, LS, PRH, and FSP supervised the study. All authors contributed to manuscript revision, read, and approved the submitted version.

## Conflict of Interest

The authors declare that the research was conducted in the absence of any commercial or financial relationships that could be construed as a potential conflict of interest.

## Publisher’s Note

All claims expressed in this article are solely those of the authors and do not necessarily represent those of their affiliated organizations, or those of the publisher, the editors and the reviewers. Any product that may be evaluated in this article, or claim that may be made by its manufacturer, is not guaranteed or endorsed by the publisher.

## Footnotes

1
https://github.com/lens-biophotonics/ZetaStitcher
