# Supplementary material for: Comparison of Different Tissue Clearing Methods for Three-Dimensional Reconstruction of Human Brain Cellular Anatomy Using Advanced Imaging Techniques
Source: Front Neuroanat. 2021 Nov 11;15:752234. doi: 10.3389/fnana.2021.752234 (PMC8632656; doi:10.3389/fnana.2021.752234)
Supplement: Supplementary file 1 [file Data_Sheet_1.pdf]

Supplementary Material

1.1 Supplementary Figures

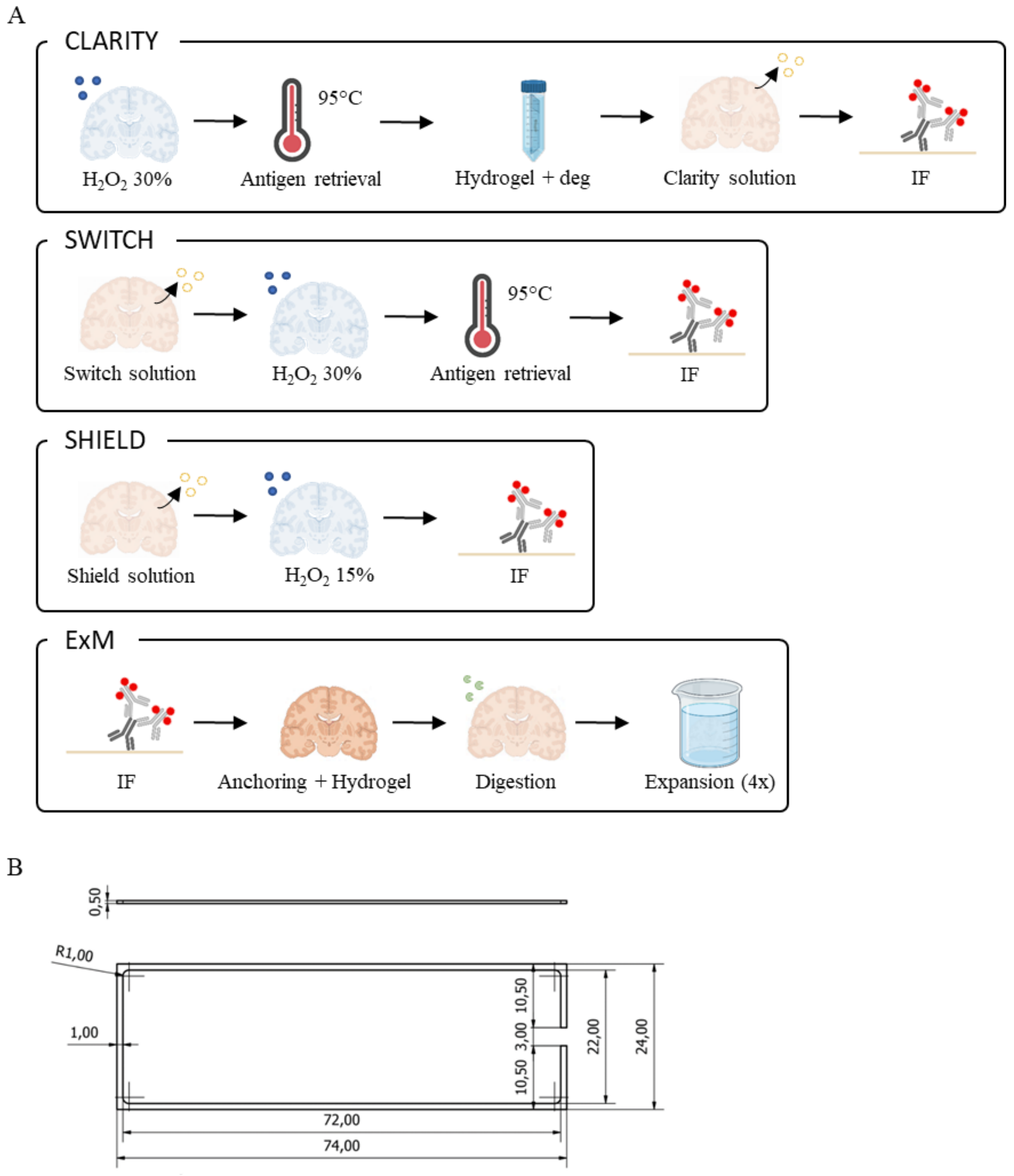

Supplementary Figure 1. (A) Schematic representations of the best protocols used for each clearing method. Created with BioRender.com. (B) Schematic drawing of the spacer used for assembling the sandwiches.

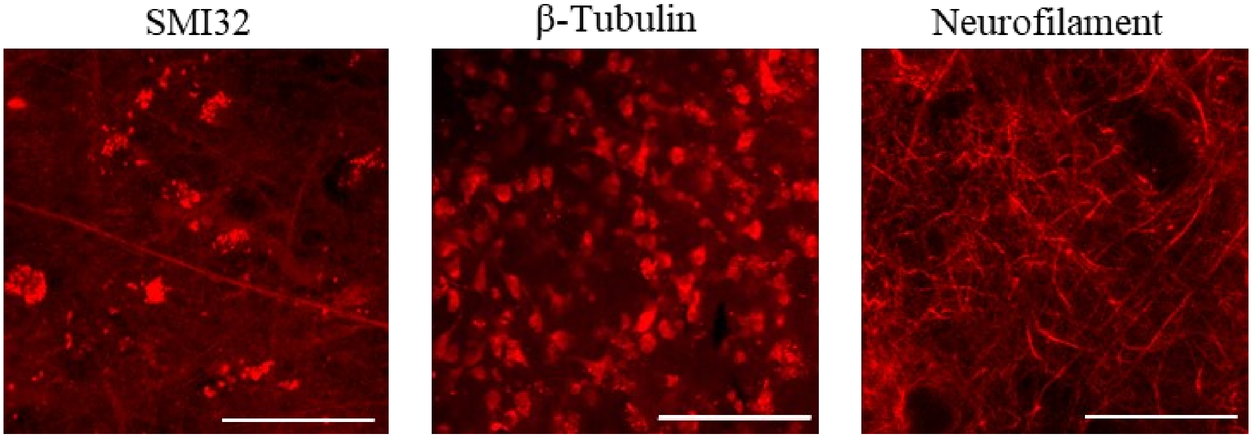

Supplementary Figure 2. Validation of the CLARITY meshgel in human brain slices. Immunofluorescent staining for additional neuronal markers of (β-Tubulin (center), and Neurofilament (right)) in CLARITY-processed human brain slices (area: superior frontal cortex). Excitation light 568 nm, laser power 1 mW. Objective lens 60x; NA 1.4. Scale bar, 50 µm.

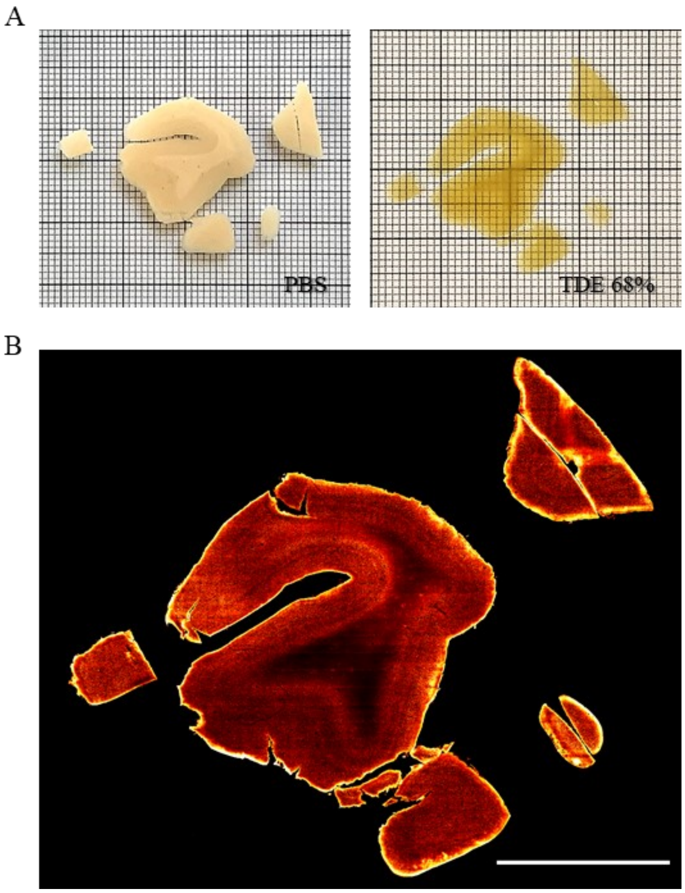

Supplementary Figure 3. Representative LSM Broca's area images. (A) Broca's area images of human brain slices in PBS (pre-clearing) and after refractive index matching in the 68% TDE/PBS solution. (B) SWITCH-processed slice stained with anti-NeuN antibody. MIP of 60 µm at 150 µm depth. Scale bar 1 cm.

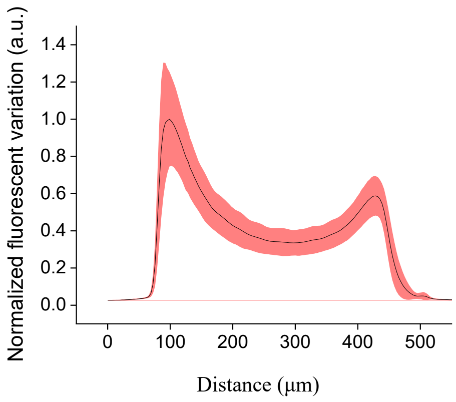

Supplementary Figure 4. Normalized plot intensity profiles of a representative stack from a 4x4x0.048 cm^2 reconstruction of human brain slab (black line: mean; light-red outline: standard deviation).

1.2 Supplementary Tables

Cat. N.	Clarity	Switch	Shield
STJ113146	No	No	No
26975-1-AP	No	Yes	Yes
ARG52283	No	No	No
ARG10712	No	Yes	yes
ab104224	No	No	No
ABN78	No	No	No
ab207282	No	No	No
ab134014	No	No	No
ABN91	No	Yes	yes
D4G40	No	No	No

Supplementary Table 1. Summary results of anti-NeuN labelling for each clearing method.
